# Splenic preservation versus splenectomy in laparoscopic distal pancreatectomy: a propensity score-matched study

**DOI:** 10.1007/s00464-019-06901-z

**Published:** 2019-06-24

**Authors:** Alma L. Moekotte, Sanne Lof, Steve A. White, Ravi Marudanayagam, Bilal Al-Sarireh, Sakhanat Rahman, Zahir Soonawalla, Mark Deakin, Somaiah Aroori, Basil Ammori, Dhanny Gomez, Gabriele Marangoni, Mohammed Abu Hilal, For the Minimally Invasive liver and Pancreatic Surgery Study Group-UK (MI-LAPS UK)

**Affiliations:** 1 Department of Surgery, University Hospital Southampton NHS Foundation Trust, Tremona Road, Southampton, SO16 2YD UK; 2 Department of Surgery, Newcastle Upon Tyne Hospitals NHS Foundation Trust, Newcastle, UK; 3 Department of Surgery, University Hospitals Birmingham NHS Foundation Trust, Birmingham, UK; 4 Department of Surgery, Morriston Hospital, Swansea, UK; 5 Department of Surgery, Royal Free London NHS Foundation Trust, London, UK; 6 Department of Surgery, Oxford University Hospitals NHS Foundation Trust, Oxford, UK; 7 Department of Surgery, Royal Stoke University Hospital, Stoke, UK; 8 Department of Surgery, Plymouth Hospitals NHS Trust, Plymouth, UK; 9 Department of Surgery, Manchester University NHS Foundation Trust, Manchester, UK; 10 Department of Surgery, Nottingham University Hospitals NHS Trust, Nottingham, UK; 11 Department of Surgery, University Hospitals Coventry and Warwickshire NHS Trust, Coventry, UK

**Keywords:** Laparoscopic distal pancreatectomy, Laparoscopic spleen-preserving distal pancreatectomy, Splenectomy, Benign, Low-grade malignant

## Abstract

**Background:**

The laparoscopic approach in distal pancreatectomy is associated with higher rates of splenic preservation compared to open surgery. Although favorable postoperative short-term outcomes have been reported in open spleen-preserving distal pancreatectomy when compared to distal pancreatectomy with splenectomy, it is unclear whether this observation applies to the laparoscopic approach. The aim of this study is to compare laparoscopic spleen-preserving distal pancreatectomy (LSPDP) with laparoscopic distal pancreatectomy with splenectomy (LDPS).

**Study design:**

This is a UK wide, propensity score-matched study, including patients who underwent LSPDP or LDPS between 2006 and 2016. Short-term outcomes were compared between LSPDP and LDPS according to intention to treat. Additionally, risk factors for unplanned splenectomy were explored.

**Results:**

A total of 456 patients were included from eleven centers (229 LSPDP and 227 LDPS). We were able to match 173 LSPDP cases to 173 LDPS cases, according to intention to treat. No differences were seen in postoperative morbidity between the groups. The only identified risk factor for unplanned splenectomy was tumor size ≥ 30 mm.

**Conclusions:**

Preserving the spleen during laparoscopic distal pancreatectomy is not associated with a lower postoperative morbidity compared to sacrificing the spleen. Tumor size is a risk factor for unplanned splenectomy.

Splenic preservation has been advocated in patients undergoing distal pancreatectomy for benign or low-grade malignant lesions because of its hematological and immunological advantages [1–4]. The two most commonly used spleen-preserving techniques are the splenic vessel preservation (SVP), where the splenic artery and vein are preserved [2]; and the Warshaw technique (WT), where the splenic artery and vein are ligated and perfusion of the spleen is maintained by the short gastric and the left gastroepiploic vessels [1].

Historically, distal pancreatectomy has been carried out with concomitant splenectomy as a result of the spleen having anatomical proximity to, and sharing principal vessels with, the left pancreas [5]. However, splenectomy comes with certain consequences; patients are at increased risk of developing severe septic complications such as overwhelming post-splenectomy infection (OPSI) syndrome [6], thromboembolic events [7] as well as developing certain malignancies [8, 9]. Moreover, two meta-analyses have shown favorable short-term outcomes in spleen-preserving distal pancreatectomy compared with distal pancreatectomy with splenectomy [10, 11]. However, the vast majority of patients included in these meta-analyses underwent open surgery. Nowadays, laparoscopic distal pancreatectomy has become the preferred approach, especially for benign and low-grade malignant lesions [12] [13]. There are a limited number of studies comparing laparoscopic spleen-preserving distal pancreatectomy (LSPDP) with laparoscopic distal pancreatectomy with splenectomy (LDPS) and the reported results on postoperative morbidity are inconsistent [14–18]. Moreover, outcomes are likely to be impacted by treatment allocation bias due to the retrospective nature of the studies and the conversion from LSPDP to LDPS due to intraoperative events.

The aims of this study are to compare short-term outcomes after LSPDP and LDPS in patients with benign and low-grade malignant lesions of the distal pancreas, using intention-to-treat analysis and propensity score matching, as well as to explore risk factors of unplanned splenectomy in intended LSPDP.

## Methods

### Study design

This retrospective cohort study was performed among eleven tertiary referral centers throughout the UK. Data were collected from consecutive patients who underwent LSPDP or LDPS between February 2006 and December 2016. The eligibility for splenic preservation was judged by a multi-disciplinary team (MDT) based on radiological and histological characteristics of the lesion and surgeons’ experience. Indications for splenectomy included suspected pancreatic ductal adenocarcinoma (PDAC), suspected lymphoma, presence of concomitant splenic vein thrombosis, suspected MCN larger than 4 cm, lesions in close proximity to the splenic hilum and NET with a high Ki67 index. All patients with definitive diagnosis of PDAC and lymphoma were excluded from analysis. Patients were analyzed according to intention to treat.

Our study was based on an anonymized database, therefore, ethical review was not required according to Health Research Authority (HRA) regulations. According to the HRA, both Research Ethics Committee (REC) and HRA approval are not required for research databases, this includes the release of non-identifiable data for analysis. Due to the retrospective nature of the study, written informed consent was not obtained.

### Outcome measures

Demographics and tumor characteristics included age, sex, tumor size, and histopathologic diagnosis. Perioperative outcome measures were operative time (OT), estimated blood loss (EBL), conversion to laparotomy, extended distal pancreatectomy [as defined by the International Study Group on Pancreatic Surgery (ISGPS)] [19], postoperative blood transfusion and number of units, length of hospital stay (LOS), reoperation, 30-day morbidity, and 30-day mortality. Complications were classified according to the Clavien–Dindo classification, major complications were defined as 3a or above [20]. Postoperative pancreatic fistulas (POPF) were defined and classified according to the definition of the ISGPS [21]. POPF grade A was considered an asymptomatic biochemical leak and not counted as a complication, according to the modifications of the ISGPS definition of POPF [22].

### Surgical procedure

Similar surgical techniques were used in all centers with minor variation in regard to patient’s and surgeon’s position. The detailed procedure has been previously described by some of our authors [23–26]. A similar technique was adopted in all centers. In brief, five ports (three 5-mm ports and two 10/12-mm ports) were used. After accessing the lesser sac, intraoperative ultrasound was performed if deemed necessary by the performing surgeon and pancreatic dissection was started using an ultrasonic or bipolar dissector by mobilizing the lower pancreatic margin and gaining access to the posterior pancreatic surface. A tape was placed around the pancreas and lifted to expose surgical planes. The pancreas was divided by using an endoscopic stapler (Echelon 60, Ethicon EndoSurgery, Cincinnati, OH or Tri-Staple™, Medtronic). In cases of LDPS, the spleen was mobilized to be retrieved en-block with the pancreas. The specimen was removed through a Pfannenstiel incision using an impermeable bag (Endocatch, Ethicon EndoSurgery). The two most commonly used spleen-preserving techniques were SVP, as described by Kimura and colleagues [2] and the WT [1]. In general, SVP was performed in those without evidence of vascular involvement. Any need to perform Warshaw technique procedure was due to the unexpected need to divide a vessel. The viability of the spleen was assessed based on the visual aspect of the spleen.

All patients who were planned to undergo a splenectomy were vaccinated preoperatively, if time permitted. If not, vaccination took place on 2 weeks postoperatively. Patients who were planned for LSPDP were not vaccinated preoperatively.

### Propensity score matching

To minimize the impact of treatment allocation bias, patients who were planned to undergo LSPDP were matched to patients who were planned for LDPS, using propensity scores. Propensity scores were based on the following baseline variables: age, sex, and tumor size. Matching was performed on a nearest neighbor basis, in a 1:1 ratio without replacement and with a caliper width of 0.01. Balance was assessed using the standardized mean difference (SMD). Optimal balance is achieved when SMD is 0.1 or below.

### Statistical analysis

Normally distributed continuous variables are expressed as means with standard deviation (SD). Non-normally distributed variables are reported as medians with interquartile ranges (IQR) or full ranges. Outcomes were compared using the independent samples *t* test for normally distributed variables. The Mann–Whitney *U* test was used to compare non-normally distributed variables. Categorical variables were compared by Chi-square or Fisher’s exact test as appropriate. Logistic regression was carried out to identify risk factors for unplanned splenectomy; variables with a *p*-value < 0.2 in univariable analysis were subsequently entered in a multivariable logistic regression. A two-tailed *p*-value < 0.05 was considered statistically significant. Data were analyzed using SPSS^®^ 24.0 software (SPSS, Chicago, IL, USA).

## Results

### Patient characteristics

**Fig. 1:**
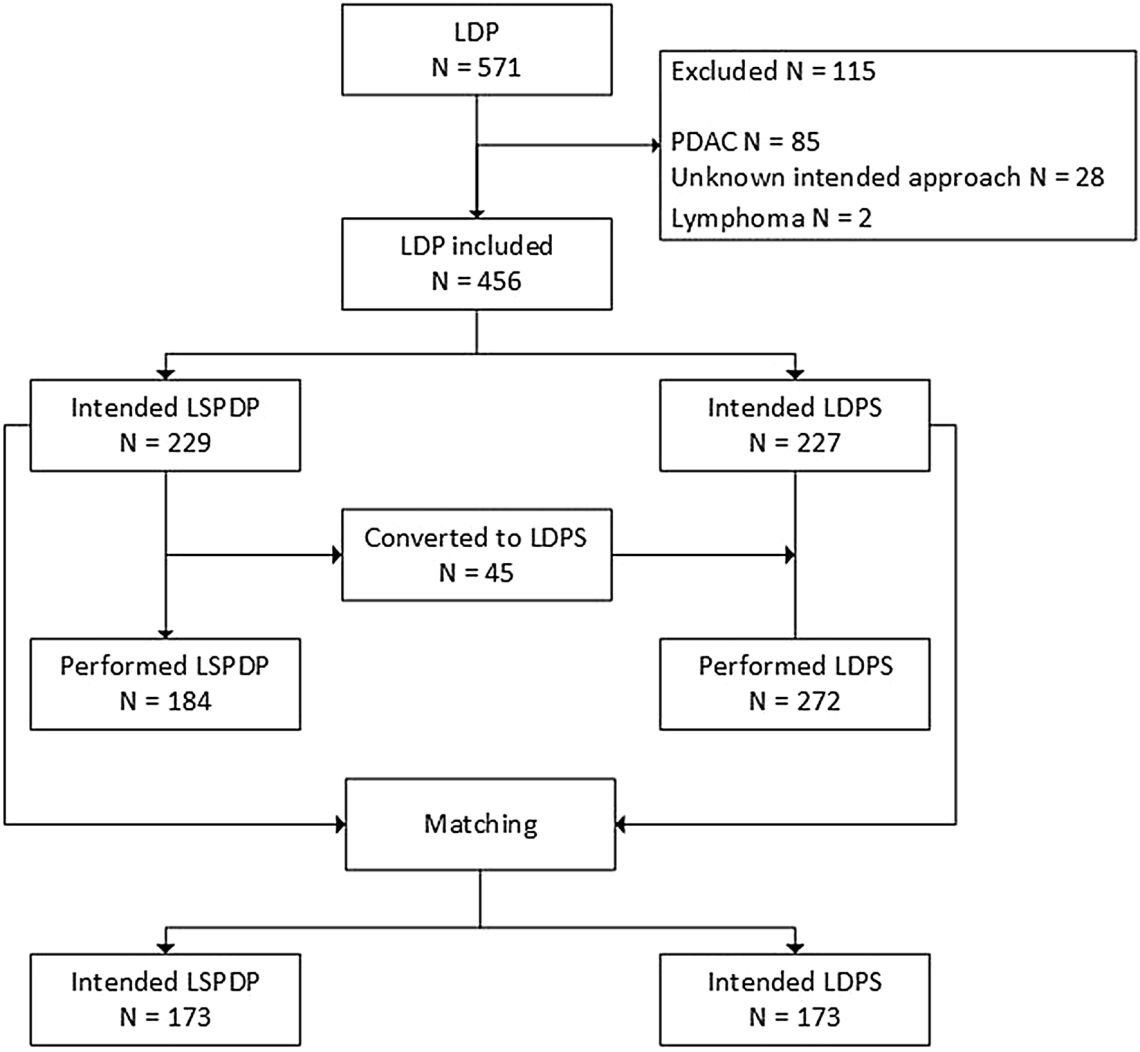
Study flowchart. *LDP* Laparoscopic distal pancreatectomy, *LSPDP* Laparoscopic spleen-preserving distal pancreatectomy, *PDAC* Pancreatic ductal adenocarcinoma

In total, 571 patients underwent laparoscopic distal pancreatectomy during the study period. Overall, 115 patients were excluded; 85 patients with PDAC, two with lymphoma, and 28 because the intended approach (spleen preserving or splenectomy) was not reported. The remaining 456 patients were included for analysis. The mean age of the cohort was 56 ± 16 years old and 293 (64%) were female. The most common histopathologic diagnoses were neuroendocrine tumor (NET), mucinous cystic neoplasm (MCN), and intraductal papillary mucinous neoplasm (IPMN). LSPDP was attempted in 229 patients, in whom splenic preservation was achieved in 184 (80%), some 45 (20%) were converted to LDPS. Of the 184 LSPDP, 124 were performed according to SVP [2] and 60 according to the Warshaw technique [1]. A total of 227 patients were planned to undergo LDPS, an additional 45 patients underwent unplanned splenectomy, therefore the total number of patients in whom LDPS was performed was 272. We were able to match 173 cases of intended LSPDP to 173 cases of intended LDPS. Of those 173 LSPDP, 150 patients underwent splenic preservation; SVP in 101 patients and the Warshaw technique in 49 patients. Figure 1 shows the study flowchart of the study.

Baseline characteristics of LSPDP and LDPS before propensity score matching are shown in Table 1. There was unbalance between the two groups in terms of age (55 ± 16 years in LSPDP and 57 ± 16 years in LDPS, SMD = 0.13), sex (67% female in LSPDP and 61% female in LDPS, SMD = 0.14), and tumor size (30 ± 21 mm versus 37 ± 25 mm in LSPDP and LDPS, SMD = 0.30). Table 2 presents the baseline characteristics after propensity score matching, which shows there is optimal balance in terms of sex (63% and 65% female in LSPDP and LDPS, respectively, SMD = 0.05) and tumor size (32 ± 21 mm in LSPDP and 33 ± 22 mm in LDPS, SMD = 0.05. For age, optimal balance was not reached (58 ± 15 years in LSPDP and 56 ± 16 years in LDPS, SMD = 0.13).

**Table 1:** Patient and tumor characteristics before matching

	LSPDP (*N* = 229)	LDPS (*N* = 227)	*P*-value	SMD
Age, years (SD)	55 (16)	57 (16)	0.21	0.13
Female, *n* (%)	154 (67)	139 (61)	0.18	0.14
Tumor size, mm (IQR)	30 (21)	37 (25)	0.002	0.30
Histology, *n* (%)				
NET	87 (38)	70 (31)		
MCN	50 (22)	63 (28)		
SCN	27 (12)	13 (6)		
IPMN	21 (9)	39 (17)		
Chronic pancreatitis/pseudocyst	13 (5.5)	22 (10)		
Other cyst	7 (3)	6 (2.5)		
RCC metastasis	7 (3)	3 (1)		
SPT	6 (2.5)	6 (2.5)		
No tumor	4 (1.5)	–		
Other metastasis	2 (1)	3 (1)		
GIST	2 (1)	2 (1)		
Other	2 (1)	–		
Acinar cell carcinoma	1 (0.5)	–		

*LSPDP* laparoscopic spleen-preserving distal pancreatectomy, *LDPS* laparoscopic distal pancreatectomy with splenectomy, *SMD* standardized mean difference, *NET* neuroendocrine tumor, *MCN* mucinous cystic neoplasm, *SCN* serous cystic neoplasm, *IPMN* intraductal papillary mucinous neoplasm, *RCC* renal cell carcinoma, *SPT* solid papillary tumor

**Table 2:** Patient and tumor characteristics after matching

	LSPDP (N = 173)	LDPS (N = 173)	*P*-value	SMD
Age, years (SD)	58 (15)	56 (16)	0.26	0.13
Female, *n* (%)	109 (63)	112 (65)	0.74	0.05
Tumor size, mm (SD)	32 (21)	33 (22)	0.55	0.05
Histology, *n* (%)				
NET	66 (38)	57 (33)		
MCN	43 (25)	51 (29)		
SCN	20 (11.5)	8 (5)		
IPMN	20 (11.5)	29 (17)		
Chronic pancreatitis/pseudocyst	8 (5)	14 (8)		
Other cyst	7 (4)	5 (3)		
RCC metastasis	3 (2)	1 (0.5)		
SPT	–	5 (3)		
No tumor	1 (0.5)	–		
Other metastasis	2 (1)	3 (1.5)		
Other	1 (0.5)	–		
GIST	1 (0.5)	–		
Acinar cell carcinoma	1 (0.5)	–		

*LSPDP* laparoscopic spleen-preserving distal pancreatectomy, *LDPS* laparoscopic distal pancreatectomy with splenectomy, *SMD* standardized mean difference, *NET* neuroendocrine tumor, *MCN* mucinous cystic neoplasm, *SCN* serous cystic neoplasm, *IPMN* intraductal papillary mucinous neoplasm, *RCC* renal cell carcinoma, *SPT* solid papillary tumor

### Perioperative outcomes

Table 3 shows the perioperative outcomes of LSPDP versus LDPS before propensity score matching. In the LSPDP group, the mean operative time (OT) was 26 min shorter than in LDPS, *p *= 0.002. The estimated blood loss (EBL) during LSPDP was 150 ml (100–300), which was comparable to LDPS: 200 ml (100–320), *p *= 0.12. A postoperative blood transfusion was given in 14/229 (6%) in the LSPDP group and in 21/227 (10%) in the LDPS group, *p *= 0.13. Conversion to laparotomy was required in 21/229 (9%) patients undergoing LSPDP and in 34/227 (15%) in the LDPS group, *p *= 0.06. Postoperative complication rate and the incidence of POPF grade B or C were comparable in both groups. The median length of hospital stay (LOS) was one day shorter after LSPDP than after LDPS (6 days (5–8) vs. 7 days (5 – 9), *p *= 0.01). A total of 16 patients developed some degree of splenic ischemia, of which four had undergone SVP and 12 the Warshaw technique. All 16 patients were managed conservatively.

**Table 3:** Perioperative outcomes before matching

Intraoperative outcomes	LSPDP (N = 229)	LDPS (N = 227)	*P*-value
Splenic preservation, *n* (%)	184 (80)	–	–
SVP, *n* (%)	124 (67)	–	–
Warshaw technique, *n* (%)	60 (33)	–	–
Operative time, min (SD)	231 (79)	257 (87)	0.002
Estimated blood loss, mL (IQR)	150 (100–300)	200 (100–320)	0.18
Conversion, *n* (%)	21 (9)	34 (15)	0.06
Extended distal pancreatectomy, *n* (%)	9 (4)	8 (4)	0.82

Postoperative outcomes			
Complications, *n* (%)	89 (41)	100 (45)	0.49
Major complications, *n* (%)	32 (14)	35 (16)	0.68
POPF B/C, *n* (%)	36 (16)	41 (18)	0.49
Grade			0.41
B	30	31	–
C	6	10	–
Splenic ischemia, *n* (%)	16 (7)	–	–
30-day mortality, *n* (%)	2 (1)	–	0.50
Reoperation, *n* (%)	12 (5)	9 (4)	0.50
Hospital stay, days (IQR)	6 (5–8)	7 (5–9)	0.01
Blood transfusion, *n* (%)	14 (6)	21 (10)	0.13
Units, *n* (range)	2 (1–6)	2 (1–14)	0.63

*LSPDP* laparoscopic spleen-preserving distal pancreatectomy, *LDPS* laparoscopic distal pancreatectomy with splenectomy, *SVP* splenic vessel preservation, *POPF* postoperative pancreatic fistula

Table 4 shows the demographics and tumor characteristics of LSPDP and LDPS after propensity score matching. The number of patients after matching was 173 in both groups. The OT was 20 min shorter in LSPDP (234 ± 77 min) compared to LDPS (256 ± 88 min), *p *= 0.02. EBL during LSPDP was comparable with the blood loss during LDPS. However, the proportion of patients needing a postoperative blood transfusion was less after LSPDP (4%) compared to LDPS (11%), *p *= 0.02. Conversion to laparotomy was required in a comparable number of patients undergoing LSPDP and LDPS. The postoperative complication rate and the incidence of POPF grade B or C were similar in both groups, as was the LOS. A total of 13 patients developed some degree of splenic ischemia, of which four had undergone SVP and nine the Warshaw technique. All 13 patients were managed conservatively.

**Table 4:** Perioperative outcomes after matching

Intraoperative outcomes	LSPDP (N = 173)	LDPS (N = 173)	*P*-value
Splenic preservation, *n* (%)	150 (87)	–	–
SVP, *n* (%)	101 (67)	–	–
Warshaw technique, *n* (%)	49 (33)	–	–
Operative time, min (SD)	234 (77)	256 (88)	0.02
Estimated blood loss, mL (IQR)	150 (100–270)	175 (100–315)	0.24
Conversion, *n* (%)	16 (9)	22 (13)	0.30
Extended distal pancreatectomy, *n* (%)	7 (4)	6 (4)	0.78

Postoperative outcomes			
Complications, *n* (%)	73 (42)	77 (45)	0.95
Major complications, *n* (%)	28 (17)	25 (15)	0.60
POPF B/C, *n* (%)	31 (18)	27 (16)	0.58
Grade			0.36
B	26	20	–
C	5	7	–
Splenic ischemia, *n* (%)	13 (8)	–	–
30-day mortality, *n* (%)	1 (0.5)	–	0.50
Reoperation, *n* (%)	10 (6)	6 (4)	0.29
Hospital stay, days (IQR)	6 (5–8)	7 (5–8)	0.22
Blood transfusion, *n* (%)	7 (4)	17 (11)	0.02
Units, *n* (range)	2 (1–2)	2 (1–14)	0.50

*LSPDP* laparoscopic spleen-preserving distal pancreatectomy, *LDPS* laparoscopic distal pancreatectomy with splenectomy, *SVP* splenic vessel preservation, *POPF* postoperative pancreatic fistula

### Unplanned splenectomy

Table 5 shows the univariable logistic regression of risk factors for unplanned splenectomy during intended LSPDP. Multivariable analysis was not performed as only one variable reached a *p*-value < 0.2. Therefore, the sole identified risk factor for unplanned splenectomy in intended LSPDP in this study was tumor size ≥ 30 mm (OR = 2.279 [1.16 – 4.48], *p *= 0.02).

**Table 5:** Univariable analysis of risk factors for splenectomy in intended splenic preservation

	Univariable	
	Odds ratio (95% CI)	*P*-value
Age		
≥ 60 years old	0.77 (0.39–1.51)	0.45
Sex		
Female	0.76 (0.39–1.52)	0.45
Tumor size		
≥ 30 mm	2.28 (1.16–4.48)	0.02
Extended distal pancreatectomy	2.31 (0.53–10.08)	0.26

## Discussion

In this study, splenic preservation was achieved in 80% of intended LSPDP, while splenic preservation rates in laparoscopic cohorts in the literature vary between 29 and 86% [14, 16, 18]. This variation could be explained by differences in surgeon’s experience in performing laparoscopic distal pancreatectomy, the long learning curve [27] as well as the preferred technique of splenic preservation (SVP or WT).

Differences in short-term outcomes between LSPDP and LDPS, in our intention-to-treat analysis, before matching, included a shorter OT and a shorter LOS in the LSPDP group. However, after propensity score matching only OT was confirmed to be shorter in LSPDP.

Similarly, Dai and colleagues, who performed a retrospective cohort study comparing LSPDP and LDPS using intention-to-treat analysis, reported a shorter OT and less EBL in the LSPDP group [14]. After propensity score matching, only a shorter OT in LSPDP was confirmed. This emphasizes the importance of propensity score matching in minimizing the allocation treatment bias and offering a more sound evidence on the difference between treatment modalities near to what can be achieved in a randomization setting [28].

A possible explanation for a longer OT if splenectomy is performed is the need for complete mobilization of the pancreas and its vessels, with complete dissection of the retroperitoneal reflection. Whereas, in LSPDP, this mobilization is not or only partially needed. In addition, if the spleen is not removed it does not need mobilization either, which potentially saves time. Another possible explanation for the shorter OT in LSPDP is that LSPDP might have been performed by more experienced surgeons compared to LDPS.

No differences were seen in 30-day complication rate or in the incidence of POPF, both before and after matching. Interestingly, a meta-analysis by Shi and colleagues reported less overall morbidity, fewer postoperative infections, less EBL, and a lower rate of POPF in spleen-preserving distal pancreatectomy (SPDP) compared to distal pancreatectomy with splenectomy (DPS) [10]. Another meta-analysis by He and colleagues showed a lower incidence of intra-abdominal abscesses in SPDP and a shorter LOS but no difference in overall morbidity or POPF rate, compared to DPS [11???]. The reduction of morbidity demonstrated in the aforementioned meta-analyses is not consistent with our results or with the report by Dai and colleagues. One explanation might be that the vast majority of the patients included in these meta-analyses underwent open surgery. Possibly, the laparoscopic approach has a greater favorable impact on the short-term outcomes leading less complications regardless of the splenic preservation, as it is previously demonstrated that laparoscopic distal pancreatectomy is associated with lower morbidity [29–32], shorter hospital stay, and less blood loss [12, 29, 30] compared to open distal pancreatectomy. Moreover, it is likely that treatment allocation bias played a role in these meta-analyses, as all studies included were retrospective cohort studies that analyzed cases of unplanned splenectomy in the DPS group. This group of unplanned splenectomies can greatly affect outcomes, as unplanned splenectomies are most likely to be the consequence of uncontrollable bleeding from the splenic vessels. Therefore, in the current study, unplanned splenectomies were analyzed in the LSPDP group.

Interestingly, the current study showed that a smaller proportion of patients received a postoperative blood transfusion after LSPDP than after LDPS, even though, EBL was comparable in both groups (150 ml vs. 200 ml in LSPDP and LDPS, respectively). Possibly, patients in the LDPS group were more likely to have comorbidities and therefore had a lower threshold for receiving a blood transfusion. As no parameters on preoperative fitness or comorbidities were documented in this study, this outcome should be interpret with caution.

This study has some other limitations. First, the retrospective nature could have led to treatment allocation bias, as the group of patients undergoing LSPDP was selected based on the judgement of the MDT, where the feasibility of splenic preservation could have played a major role, leading to less complex cases in the LSPDP group. However, treatment allocation bias has been minimized by performing intention-to-treat analysis and propensity score matching. Second, there might have been differences in indication for LSPDP and LDPS between the different centers. The strengths of this study are that, to the best of our knowledge, this is the largest cohort comparing LSPDP and LDPS, the second study to match these groups using propensity scores, and having very similar results to the other propensity score-matched study.

## Conclusions

A high splenic preservation rate was achieved with tumor size as a risk factor for unplanned splenectomy. Preserving the spleen during laparoscopic distal pancreatectomy is not associated with a lower postoperative morbidity compared to sacrificing the spleen. However, taking in consideration the long-term risks of post-splenectomy patients, the authors believe splenic preservation should be attempted in laparoscopic distal pancreatectomy for benign or low-grade malignant lesions as this study shows the approach is safe and feasible.

## Abbreviations

LSPDP: Laparoscopic spleen-preserving distal pancreatectomy
LDPS: Laparoscopic distal pancreatectomy with splenectomy
SVP: Splenic vessel preservation
WT: Warshaw technique
OT: Operative time
EBL: Estimated blood loss
ISGPS: International Study Group on Pancreatic Surgery
POPF: Postoperative pancreatic fistula
SD: Standard deviation
IQR: Interquartile ranges
LOS: Length of stay
